# Marriage of First Cousins

**Published:** 1900-06-16

**Authors:** 


### Marriage of First Cousins.

There are few subjects in regard to which more positive opinions have been expressed than as to the iniquity of first cousins marrying. There are few, probably, in regard to which such opinions have had less basis in fact, and as the question is constantly arising it is well that one should be ready with an answer which shall be positive and free from doubt. Everyone, of course, understands that in-breeding intensifies hereditary tendencies, and that in this way the marriage of first cousins is to be avoided where there is a bad family history. Clearly the marriage of first cousins in such a case is as bad as mating any two ordinary people each of whom has a tendency to the same disease. Worse, indeed, since in the case of cousins the "match" is so exact as to greatly increase the probability of the taint being not only transmitted but intensified. But while arguments based upon heredity may be a bar to such marriages in some families, they may actually be an encouragement to them in others. What an individual receives by heredity is derived partly from his parents, and partly from the far back ancestry of both of them, and thus any peculiarities which may crop up now and again, whether in the direction of genius or disease, are quickly wiped out by the preponderating influence of those untold generations of mediocrity which have preceded them. Thus the race, constantly tending towards the commonplace, rights itself. It is, indeed, only by repeated breeding of like with like that family peculiarities can be originated or maintained, and there can be little doubt that in some isolated communities in-breeding has been as useful an influence in producing stamina as in others it has been disastrous in leading to degeneration. So far then as heredity is concerned, such in-breeding as is involved in the marriage of first cousins is good or bad purely according as it tends to intensify a good or a bad strain, not, of course, as the strain shows itself in the individual, but in the family for generations back. But there is another side to the question, or, rather, there is another question altogether, namely, Do consanguineous marriages, from the mere fact of consanguinity, irrespective of the question of heredity, tend to produce a deteriorated offspring? To this also we may answer that there is no proof of anything of the sort. In an article in this month's *Polyclinic* it is pointed out that the traditional creed so commonly entertained by the public, and to a certain extent even by breeders themselves, that it is necessary to avoid in-and-in breeding is based upon a fallacy. It is true that if in-breeding be persisted in for too long harm may result, but this is because the peculiarity or quality which the breeder is trying to perpetuate or intensify itself partakes of the nature of disease. Then no doubt it becomes prejudicial. Of this pigs offer, perhaps, the best example. In-and in breeding of pigs if too close and too long continued, results in sterility. But the explanation is simple. Pigs are bred for fatness, and the tendency to fatten, as seen in the prize pig, is in itself a disease, and is antagonistic to fecundity. Certainly among animals in a state of nature there are no provisions for the prevention of in-and-in breeding—there are many conditions, indeed, which favour it—whilst there are no facts to show that any harm results. On the other hand, almost all valuable stocks have originated by careful in-breeding, and have been so maintained, some of them having kept up their qualities without the slightest failure in fecundity or in general vigour through long successions of years. The writer of the article above referred to, whom we take to be Mr. Jonathan Hutchinson, says that the stock-breeder never fears to bring together first cousins, and that many of the marriages which he designedly promotes are incestuous in the highest degree, yet that it is often from such that he obtains the highest class animals. Moreover, the problem as it regards humans is very much simpler than in regard to domestic animals. With the stock breeder it is a question of the influence of prolonged repetition of the same process, whereas with humans the question as it practically comes before us is concerned simply with one individual marriage in one particular generation. If ever harm has seemed to follow consanguineous mating among animals it has arisen from a long series, not from an individual union. The answer to the question must then be that, if in the family in question there be a definite tendency to such diseases as tuberculosis or cancer or insanity, there is a risk that this tendency may be intensified by the marriage of first cousins, while if the family history be a good one there may be greater security in such a marriage than in one with a stranger, about whose antecedents less may be known. Nor must the known existence of some amount of taint be held an absolute bar unless the alternative be also known, and be free from any such taint. "Nothing is easier than in such a case to make a leap from the pan into the fire, and a parent who forbids a first-cousin marriage because an ancestor has been in an asylum or died of cancer, must face the responsibility of being not improbably the indirect cause of some yet more risky connection." Too much caution may over-reach itself.

